# Promoting an ethic of engagement in pediatric palliative care research

**DOI:** 10.1186/s12904-015-0048-5

**Published:** 2015-10-16

**Authors:** Vasiliki Rahimzadeh, Gillian Bartlett, Cristina Longo, Laura Crimi, Mary Ellen Macdonald, Nada Jabado, Carolyn Ells

**Affiliations:** Department of Family Medicine, Centre of Genomics and Policy, McGill University, 5858 Côte-des-Neiges, Suite 300, Montréal, QC H3S 1Z1 Canada; Department of Family Medicine, McGill University, 5858 Côte-des-Neiges, Suite 300, Montréal, QC H3S 1Z1 Canada; Division of Oral Health and Society, Pediatric Palliative Care Research, Montreal Children’s Hospital, McGill University, #530-2001 McGill College Avenue, Montreal, QC H3A 1G1 Canada; Department of Pediatrics, Montreal Children’s Hospital Research Institute, McGill University Health Center, 1001 Décarie Boulevard, Montreal, H4A 3J1 QC Canada; Biomedical Ethics Unit, McGill University, 3647 Peel St, Montreal, QC H3A 1X1 Canada

**Keywords:** Pediatric palliative care, Engagement, Qualitative research, Ethics, Inclusion, Participation

## Abstract

**Background:**

This paper defends the ethical and empirical significance of direct engagement with terminally ill children and adolescents in PPC research on health-related quality of life. Clinical trials and other forms of health research have resulted in tremendous progress for improving clinical outcomes among children and adolescents diagnosed with a life-threatening illness. Less attention has been paid, however, to engaging this patient population directly in studies aimed at optimizing health-related quality of life in PPC. Though not restricted to care at the end of life, PPC—and by extension PPC research—is in part dependent on recognizing the social complexities of death and dying and where health-related quality of life is a fundamental element. To explore these complexities in depth requires partnership with terminally ill children and adolescents, and acknowledgement of their active social and moral agency in research.

**Discussion:**

Principles of pediatric research ethics, theoretical tenets of the “new sociology of the child(hood),” and human rights codified in the United Nations Convention on the Rights of the Child (UNCRC) underpin the position that a more engagement-centered approach is needed in PPC research. The ethics, sociologies and human rights of engagement will each be discussed as they relate to research with terminally ill children and adolescents in PPC. Qualitative method(ologies) presented in this paper, such as deliberative stakeholder consultations and phenomenology of practice can serve as meaningful vehicles for achieving i) participation among terminally ill children and adolescents; ii) evidence-bases for PPC best practices; and iii) fulfillment of research ethics principles.

**Conclusion:**

PPC research based on direct engagement with PPC patients better reflects their unique expertise and social epistemologies of terminal illness. Such an approach to research would strengthen both the ethical and methodological soundness of HRQoL inquiry in PPC.

## Background

According to the American Academy of Pediatrics, the “goal of pediatric palliative care is to add life to the child’s years, not simply add years to the child’s life” [1]. Promoting a research agenda that makes central the participation of terminally ill children and adolescents in pediatric palliative care (PPC) is needed to ensure the former, and to improve PPC services in line with their lived experiences. While the focus of this paper will be PPC research at the end of life, PPC delivery is not restricted to this point in the care trajectory. Rather, it is well established that PPC should be integrated as early as possible in the care process [2]. Despite global recognition of the need for enhancing the availability and accessibility of PPC, systematic reviews of the literature reveal unmet needs in both developed and developing countries [3-5]. In response, concerted efforts to expand PPC  has enabled i) better identification of eligible patient populations; ii) greater attention to symptom control needs at the end of life,; iii) empirical research investigating health-related quality of life (HRQoL) [6, 7] and iv) evaluation of cost-benefit outcomes [8–10].

Research with terminally ill children and adolescents should therefore also parallel the service expansion of PPC in order to meet demands: “Research involving children and their families occupies a small niche in the world of research in palliative and end-of-life care, which itself is small in comparison to other areas of clinical and health services research” [11]. Considering that healthcare professionals have an ethical duty to palliate when a child is believed to be suffering at the end of life [12, 13], the lacuna of evidence-based care practices derived from PPC research with patients can be both ethically questionable and methodologically unsound [14]. To this end, HRQoL inquiry with terminally ill children and adolescents has been identified as an important, albeit under-researched domain in PPC [6, 15].

HRQoL is a socioculturally complex phenomena that has been used extensively in palliative care research to inform best practices [16, 17]. Yet, “Few reliable, valid, and developmentally appropriate methods are available for measuring the suffering and quality of life of children with life-threatening illness…” [18]. The intensely personal nature of HRQoL necessitates that research investigating its influence on PPC delivery engage participants directly as a rule whenever possible.^1^ Although greater engagement of terminally ill children and adolescents is emerging as a new priority in PPC research [4, 19], limited attention has been paid to their illness experience(s) in PPC for quality of care improvement [20]. Most studies investigating HRQoL among terminally or critically ill children and adolescents overwhelmingly represents the perspectives of parental and/or health professional proxies [21–25]. A recent study of parents’ ability to represent the ‘voice of the child’ in end of life decision-making found, however, that parents often have “difficulty in gaining an insight into their child’s perspective even though they may be intensely involved in his or her care and support during the [end of life] phase” [26]. Where children are engaged in HRQoL research within other pediatric health domains, the level of agreement between self- and proxy-reporting yield mixed results [27–33]. The marked absence of children and adolescents in studies meant to better understand their perspectives on terminal illness [34–36] can be attributed in part to the relative rarity of such illnesses in this population [37, 38]; misconception surrounding the care goals of PPC [39]; a shortage of PPC specialty training [40–42]; and stringent regulations governing the involvement of children in research [43]. Matza et al. nuance the challenges of assessing HRQoL in this population, specifically: “As more pediatric clinical trials have been initiated, however, researchers have encountered a unique set of challenges involved in assessing HRQL among children, such as identifying the age at which children can reliably report various domains of HRQL and determining whether children or their parents are the best respondents” [44].

Caring for terminally ill children and adolescents should be reflective of the complex social dimensions of death and dying patients themselves face if PPC is to be truly patient-centered [6]. Underrepresentation of this crucial stakeholder population (terminally ill children and adolescents) can misguide the development of best practices and fall short of fulfilling the ethical mandates for respect persons and justice, among others. A recent proposal of a ‘charter for the rights of the dying child’ attests to this: “To have access to child-specific palliative-care programmes that avoid futile or excessively burdensome practices and therapeutic abandonment” [45]. Engaging children and adolescents in PPC research enhances their position as a critical stakeholder community for whom effective service delivery should be designed. Pursuant to this goal, the United Nations Convention on the Rights of the Child (UNCRC) and new social theories on the child(hood) can inform the ethical frameworks upon which increased pediatric participation in PPC research can build. Each of these frameworks will be discussed in turn in the subsequent sections.

### Social theories and applicability to PPC

Research with terminally ill children and adolescents can accentuate the classical tensions that ethics review committees and researchers consider when involving minors in research. This tension follows that children warrant special protections due to their unique situation(s) of vulnerability, yet should not be categorically excluded from research that could lead to important health(care) advancements for them. Promoting research with—as opposed to on—children is inspired in part by “new sociologies of the child” [46]. The concepts articulated in the new sociology of the child, herein referred to as the new sociology, and those later codified in the UNCRC support the greater inclusion of terminally ill children and adolescents in PPC research.

Of the four guiding principles outlined in the UNCRC, respect for the views of the child under Article 12 lends the strongest support of children’s inclusion and participation in (health) research broadly [47]:

States Parties shall assure to the child who is capable of forming his or her own views the right to express those views freely in all matters affecting the child, the views of the child being given due weight in accordance with the age and maturity of the child.

While Article 12 is one of the most celebrated achievements of the UNCRC, it has also garnered the most controversy [48]. Article 12 explicitly safeguards participatory rights granted to children and adolescents, underscoring the prioritization and legal legitimacy of their voice. It is groundbreaking, “not only for what it says, but because it recognizes the child as a full human being with integrity, personality and the ability to participate freely in society” [49].

Taken together, the UNCRC and the new sociology share many theoretical underpinnings that substantiate a rights-based approach to child participation and protection. Lavalette and Cunningham summarize the central tenets of the new sociology:

1. Childhood is not merely a biological phenomenon, but a social construction, affected and shaped by wider social and cultural elements, within concrete, historical circumstances;
2. Children occupy and conduct themselves in worlds that are full of meaning for them, but about which adults are, at least partially, ignorant. It has led to an emphasis on listening to children’s voices;
3. Politically, children are powerless and disadvantaged. The new sociology is a theory of advocacy, sociology for children rather than sociology of children. This approach has closely tied into children’s rights agenda; and
4. Children are an identifiable social group, with a common [basic] set of needs and rights [50].

Each tenet will be discussed in turn as they relate to existing pediatric research ethics norms and the UNCRC.

### An ethical justification of PPC for children, by children

First, societal determinations of ‘childhood’ and ‘adulthood’ are largely the result of physiological and psychological benchmarking. Taking from early child development theory according to Piaget, maturity (and hence autonomous decision-making) surrounding death and dying is presumed to evolve chronologically with age [51]. Maturity is legally recognized to reach full development at the age of majority, 18 in most Western jurisdictions. This accepted linearity of a child’s capacity to make informed decisions is the basis upon which legal standards of a child’s capacity to consent to research (and care) are premised [52]. The primary rationale for an age of majority is then to categorically define the point when a child is capable of making informed decisions, such as the ability to consent to research. Diagnosis of a terminal illness and the pending outcome of death, however, truncates the burgeoning process of autonomy [51, 53]. Placing the same age of majority restrictions on PPC patients’ ability to participate in HRQoL research can be methodologically inappropriate for what is often considered minimal risk, qualitative research. This is particularly the case when recruitment of the research population of interest is critical to addressing knowledge gaps regarding HRQoL for terminally ill children and adolescents receiving PPC. Prevailing assumptions of when a minor reaches full decision-making ability and the level of vulnerability they experience as a result can be (unjustified) barriers to involvement in HRQoL research when taking into account the limited timeframe in which they can participate.^2^

Second, studies investigating child health, including at the end of life, cannot extrapolate findings from adult studies. Just as HRQoL assessment tools have been created specifically for use in pediatric populations, HRQoL research in pediatric palliative care should also be population-specific: “To identify practices that affect the quality of life experienced by a child with a life-threatening medical problem requires measurement tools that can reliably and validly reflect the child’s experiences, particularly when the problem has reached an advanced stage and death is expected or possible in the foreseeable future” [11]. Despite widespread clinical interest, there HRQoL infrequently engages children and adolescents directly in the development of PPC best care practices [54–56].

Moreover, the heterogeneity of ‘childhoods’, family contexts and end-of-life beliefs necessitate thoughtful consideration of research methods that can best describe the worlds children occupy. From a methodological standpoint, researcher reflexivity and coherent study design are critical for meeting bioethical mandates as well. As such, lending primacy to children and adolescents’ voices is one avenue for respecting their agency in healthcare settings, including in PPC.^3^ Hart is perhaps best known for his work in this field, describing how participation is stepwise on a ladder that culminates in an ideal empowerment model. Hart loosely defines participation as referring to “the process of sharing decisions which affect one’s life and the life of the community in which one lives…Participation is the fundamental right of citizenship” [57].

Third, in line with the ideas of participation Hart proposes, the new sociology’s emphasis on power can encourage a discussion of social and distributive justice for the inclusion of terminally ill children and adolescents in PPC research. Canada’s *Tri Council Policy Statement: Ethical Conduct for Research Involving Humans* (TCPS 2) speaks to this point directly:

The principle of Justice holds that particular individuals, groups or communities should neither bear an unfair share of the direct burdens of participating in research, nor should they be unfairly excluded from the potential benefits of research participation…Over-protectionist attitudes or practices of researchers or REBs, whether intentional or inadvertent, can exclude some members of society from participating in research…The inclusion of the young and the elderly in research, for example, ensures that treatments frequently given to these populations are effective and safe [58].

### Terms of engagement in research practice

Certainly, a commitment to research engagement with children and adolescents demands methodological consideration. Some scholars have argued the ethics of PPC research compounds the already pressing concerns regarding vulnerability in research with children generally; others deem research in palliative care *ipso facto* unethical [59]. We posit that neither children’s terminal illnesses nor the extent of their (presumed) vulnerability as a result should categorically prevent research in this area. Rapoport shares this view, urging researchers that, “The imperative to practice evidence-based medicine and strive toward optimal patient care compels us to find ways to overcome obstacles to conducting research on vulnerable patients, not by avoidance, but by confronting them directly” [41].

Engagement can therefore take on a variety of forms and depths in research practice. Ensuring assent is obtained from pediatric participants is a requisite first step. Although there is no consensus on a definition, nor the exact circumstances under which assent should be sought from children or adolescents, it is nevertheless the foundation of ethical engagement [60]. Democratic and non-tokenistic inclusion underlines the notion of participation Hart proposes, but is worth mentioning this norm is more widely accepted in Western contexts. A number of participatory methods have been recommended to overcome the ethical challenges of avoiding tokenistic involvement of children and adolescents in research [61] and other methods for HRQoL research, specifically [62].

Two methodological methods and approaches are proposed to complement the deeply personal nature of HRQoL inquiry with terminally ill children and adolescents. A research team comprised of authors VR, GB, LC and CL, in partnership with the International Childhood Astrocytoma iNtegrated Genomic and Epigenomic (iCHANGE) Consortium is piloting a deliberative stakeholder consultation method to explore facilitators and barriers to HRQoL among children and adolescents with terminal brain tumors (Longo C, Bartlett G, Rahimzadeh V, Crimi L. Deliberative Stakeholder Consultations: Giving Vulnerable Patients a Voice in Genetic Research. Forthcoming. [63]). Contrary to focus groups and other similar qualitative approaches such as citizen juries, the deliberative stakeholder consultation limits researcher mediation [64]. It aims to mitigating power differentials between researchers and participants that have been the source of considerable debate in both the health research [65–67] and bioethics communities. The hallmark of deliberative methods, such as those we use to engage terminally ill children and adolescents in our ongoing study, reflects the idea that “deliberation is more than merely a discussion of the issues. Emphasis is also given to the product that arises from discussion (e.g., a decision or set of recommendations), and the process through which that product comes about” [68].

With equal emphasis placed on understanding through engagement, a phenomenology of practice approach provides further methodological guidance for HRQoL inquiry with PPC patients. van Manen writes the primary aim of a phenomenology of practice-inspired research strives, “…to open up possibilities for creating formative relations between being and acting, between who we are and how we act, between thoughtfulness and tact” [69]. A phenomenology of practice in PPC research would give precedence to the theoretical dimensions of HRQoL as defined by PPC patients themselves.

## Conclusion

An ethic of engagement in PPC research defended in this paper activates the rights to free expression and participation of the UNCRC. It advocates for  the involvement of children and adolescents in research meant to better inform PPC delivery; improves care for future patients; promotes accessibility and availability of PPC services that are reflective of children’s experiences at the end of life; expands modes of representation among the PPC population in health research; and achieves direct stakeholder engagement with primary users of PPC services. Drawing on ethical and methodological arguments reinforced by the new sociology of the child(hood) and qualitative research, respectively, the authors propose the engagement of children and adolescents is central to augmenting care capacities in PPC.

There is yet so much to learn from children *of* the death and dying process in order to better care for future children *during* the death and dying process. The continued underrepresentation of terminally ill children and adolescents in PPC research, however, fails to ground modalities of care in the unique and evolving clinical realities they face as PPC patients. Because ensuring an optimal HRQoL is the goal of PPC, it is an ethical and methodological imperative that research in this field is inclusive of primary stakeholder populations, namely PPC patients. The deliberative stakeholder consultation and phenomenology of practice approach are two methodological decisions that can help PPC researchers achieve such inclusion. While the former has been used to engage stakeholders in other domains of health research [70], assessment of its methodological utility for exploring HRQoL in the PPC research context is currently underway.

How research engagement with PPC patients will be affected by shifts in conceptions of the child(hood) remain to be seen. But, “Ultimately the children’s rights project is not just about making a better world for children, it is about making a better world for all of us” [71]. Part of granting greater legitimacy to children’s moral and social agency, particularly in the face of terminal illness, is rendering their continued absence in PPC an antiquated protectionist argument of the past. Doing so can make way for more creative and inclusive methods in PPC research that indeed “add life to a child’s years.” The spirit of engagement promulgated by the deliberative stakeholder consultation method could also be transferable to improving understanding of HRQoL for adults in similar situations of vulnerability, namely elderly patients living with dementia [72].

The year 2014 marked the 25th anniversary of the UNCRC’s adoption, and was coined the “Year of Innovation for Equity.” In addition to encouraging novel approaches to equity, it gives us pause to reflect on the victories of the UNCRC, and spotlight areas for improvement in furthering the rights and welfare of children and adolescents worldwide. Healthcare is an ideal setting in which to strive towards realizing the ideals of participation, protection and provision the UNCRC espouses as it invokes the most basic human right afforded to all: the right to health. The emergence of international initiatives in PPC research, and identification of its pressing need in the pediatric community is one of many victories.

## Abbreviations

PPC: Pediatric palliative care
HRQoL: Health-related quality of life
UNCRC: United Nations Convention on the Rights of the Child
TCPS 2: Tri Council Policy Statement: Ethical Conduct for Research Involving Humans, 2nd edition

## Footnotes

1 Some circumstances complicate children’s ability to participate in research, to be sure. This can be particularly true for research using language-based methods (such as in qualitative research) with infants, children who are otherwise nonverbal, or those who suffer from severe cognitive impairment.

2 The ethical complexities concerning mature minors and clinical decision-making typify this case scenario. A number of studies have sought to gauge the level of decision-making ability and maturity that minors demonstrate in the face of critical illness (see for example Miller et al. [73]) where findings indicate some adolescents exhibit abilities that match, or surpass, those of consenting adults. On this point, there is some consensus in the adolescent psychology literature that suggest minors appeal to morality and logic in informing their decisions by middle adolescence [74].

3 It is worth noting that some scholars argue listening to children’s voices alone is not sufficient to promote due representation [71] or can be tokenistic if not followed up with measurable action [57, 75].
